# Metoclopramide for Acute Migraine Treatment in the Emergency Department: An Effective Alternative to Opioids

**DOI:** 10.7759/cureus.1181

**Published:** 2017-04-20

**Authors:** Mejdi Najjar, Tyler Hall, Blanca Estupinan

**Affiliations:** 1 College of Medicine, University of Central Florida College of Medicine

**Keywords:** acute migraine, metoclopramide, opioids, emergency medicine, headache

## Abstract

In light of recent warnings by the United States (US) Surgeon General and Centers for Disease Control (CDC) guidelines for recommending more prudent use of opioid narcotics, the search for a non-opioid alternative for aborting acute migraines is particularly relevant. The CDC also estimates the prevalence of opioid dependence may be as high as 26% among patients prescribed opioids for chronic pain, not due to cancer, in the primary care setting. Given such staggering data, it is imperative that we, as caretakers, not foster opioid dependence but rather continue to investigate non-opioid therapies for the management of acute migraines in the emergent care settings. Our literature review demonstrates that metoclopramide should be used more frequently as first-line therapy for an acute migraine over opioids. The use of opioids specifically has been discouraged as migraine treatment by the American Headache Society citing “insufficient evidence” as the main reason. Metoclopramide, specifically using the 10 mg dose, has been cited as “highly likely to be effective” by the same guidelines. Another major issue with opioids is the growing potential for abuse, thus minimizing the use of these drugs for only special circumstances would be beneficial overall.

## Introduction and background

Migraine headaches are extremely common in the general population, affecting an estimated 13% of all adults in the United States (US) [1]. Younger adults tend to be most affected by migraines, with peak prevalence between 30 and 49 years of age [1]. Classically, migraines present as severe, throbbing headaches, which are episodic in nature and persist between four and 72 hours [1]. In addition, patients frequently report associated symptoms, such as nausea, vomiting, photophobia, etc. Some migraine sufferers report experiencing an aura prior to onset, but symptoms and presentations are highly variable between individuals. Unsurprisingly, given the nature and severity of symptoms, acute headaches are one of the most common presentations to emergency departments (ED), accounting for 2-3% of all emergency room visits [2].

The cost burden of emergency care management of acute migraines is estimated at $700 million annually, with an additional $375 million spent on migraines requiring inpatient hospitalization [3]. In addition, the economic burden of migraine headaches is significant. Individuals suffering from migraine headaches miss an average of eight extra days of work per year due to migraines; sufferers also report another seven days of decreased productivity in the workplace as a result of symptoms [3]. In total, the global economic burden of migraines on employers is estimated to be $15 billion annually [3]. Despite significant spending efforts, management of migraine headaches remains suboptimal in all healthcare settings. 

Although migraine headaches are frequently encountered in the ED, no consensus currently exists in managing acute migraines [2]. According to one retrospective cohort of 811,419 migraines at 398 hospitals, as many as 16 oral and 20 parenteral agents were used as abortive therapies in the emergent care setting [4]. Furthermore, patients received zero to five abortive agents for symptomatic relief [4]. Medications affecting dopaminergic, serotonergic, and various pain pathways have all demonstrated efficacy as abortive agents; however, data has failed to demonstrate the superiority of one class of agents relative to others. This lack of objective data results in polypharmacy, increasing the risk of adverse events, in addition to the overall the cost of medical care. 

Despite the numerous agents available to physicians, multiple studies show opioids are prescribed to the majority of patients presenting to the ED with acute migraine headaches [2-4]. A retrospective cohort by Vinson, et al. examining more than 800,000 patients found that 77% of patients receiving opioids as abortive agents did not receive any non-opioid medications prior to the use of opioid narcotics [4].

One retrospective study examining 500 acute migraine patients presenting to the ED in five Canadian hospitals found opioids were prescribed as the initial medication for symptomatic relief in 60% of the cases [2]. Interestingly, the same study determined that patients who received opioids as abortive therapy were more likely to return to the same ED for subsequent headaches [2]. Whether due to the failure of treatment or drug-seeking behavior, it is evident that opioid narcotics should not be used as first-line abortive agents for acute migraines.

In light of recent warnings by the US Surgeon General and CDC guidelines for recommending the more prudent use of opioid narcotics, the search for a non-opioid alternative for aborting acute migraines is particularly relevant. A retrospective analysis by Mazer-Amirshahi, et al., examining the use of opioids for acute headache between 2001 and 2010, found the use of opioids for acute headaches in the ED actually increased from 20.6% (95%  confidence interval (CI); 18.1 - 23.4) to 35% (95% CI; 31.8 - 38.4; p = .001), while the use of antiemetic agents, such as metoclopramide, showed a non-significant decrease from 24.1% (95% CI; 19.6 - 29.2) to 23.5% (95% CI; 21.1 - 26.0; p = .08) over the last decade [5]. Among opioids, use of hydromorphone increased from 1.7% in 2001 (95% CI; 1.2 - 2.6) to 10.1% in 2010 (95% CI; 8.2 - 12.4; p < 0.001) – a 461% increase [5]. Meanwhile, deaths due to prescription opioid overdose have quadrupled in the past 15 years [6]. The CDC also estimates the prevalence of opioid dependence may be as high as 26% among patients prescribed opioids for chronic pain, not due to cancer, in the primary care setting [6]. Given such staggering data, it is imperative that we, as caretakers, not foster opioid dependence but rather continue to investigate non-opioid therapies for the management of acute migraines in the emergent care settings.

## Review

### Opioids for acute migraine treatment

Perhaps familiarity with opioids and their dosing has contributed to their continued first-line use in the ED. Though there may be a sense of security with prescribing opioids, significant adverse effects exist. All short-acting opioids carry the risk of tolerance and physical or psychological dependence [7]. Compared to matched controls with similar headache frequency, patients with migraines and opioid dependence had higher rates of depression, anxiety, and disability [8]. Recent research efforts have discovered an increased risk of serotonin syndrome with the use of opioids which can be fatal, especially if patients are prescribed selective serotonin reuptake inhibitors (SSRIs) and serotonin-norepinephrine reuptake inhibitors (SNRIs). Meperidine, a frequently prescribed opioid for headaches, and fentanyl inhibit serotonin reuptake while codeine and oxycodone increase synaptic serotonin by unclear mechanisms [9]. A review of medication classes associated with serotonin syndrome found that opioids were ranked second, after SSRI/SNRIs and before monoamine oxidase inhibitors (MAOI) and tricyclic antidepressants (TCAs) [10]. This may, in part, be due to increasing tolerance and the need for higher, more frequent dosing for pain control with opioids. Although chronic opioid therapy is associated with medication overuse headache, leading to more frequent and constant headaches, acute therapy is also associated with refractory headaches [11]. One study found that patients in status migrainosus achieved pain relief with first-line migraine medications, ketorolac and sumatriptan, only if they had not used opioids in the last six months [12]. This supports the aforementioned effects that opioids have on increasing central sensitization, leading to a persistent pronociceptive effect. Overall, the risks of opioid use in migraine headaches are difficult to justify when the efficacy and patient outcomes are inferior to migraine-specific treatments.

Recent studies have challenged the effectiveness of opioids and patient outcomes post-treatment. When compared to patients receiving first-line migraine medications, patients who received opioids as the primary treatment required higher total doses of medications (median: 5 vs. 3, p < 0.001) and were 2.7 times more likely to return to the ED within the first seven days due to relapse (odds ratio (OR) 2.7, 95% CI: 1.1 - 6.8, p = .035) [13]. A meta-analysis of randomized controlled trials discovered that meperidine, a favored opioid for migraines, relieved headache pain half as often as dihydroergotamine, a migraine-specific treatment. This study also revealed meperidine only provided relief 81% as often as antiemetic therapy [14]. Comparisons across controlled clinical trials found that metoclopramide and opioids decreased pain intensity [15]. One study conducted supports this theory as meperidine/promethazine and dihydroergotamine/metoclopramide provided patients with similar headache relief after one hour (77.2% vs. 86.2%, P = .37) [16]. Additional studies comparing different classes of migraine-specific medications and alternative treatments found that opioids were only superior in efficacy to placebo and ketorolac, a nonsteroidal anti-inflammatory agent [17]. However, compared to non-opioid therapy, opioid therapy significantly increased the length of hospital stays and recurrent emergency department visits. Regarding overall efficacy in headache treatment, opioids demonstrate more sedation, less pain relief, and increased recurrence [13].

### Metoclopramide for acute migraine treatment

Many drugs have been used for the treatment of acute migraine attacks; the antidopaminergic drugs used are metoclopramide and prochlorperazine. Both of these drugs antagonize the dopamine D2 receptor, also used to treat nausea and vomiting seen in acute migraines. In addition, metoclopramide has been noted to increase the absorption of other drugs and relieve gastric stasis [18]. With the noted combined effects, metoclopramide seems an ideal short-term therapy for acute migraine treatment in the ED.

A few studies over the years have demonstrated a significant clinical efficacy of metoclopramide in treating acute migraine attacks [19-21]. A meta-analysis conducted in one study from pooled data showed that metoclopramide led to significant reduction in headache pain (OR = 2.84, 95% CI = 1.05 to 7.68, p = .04) and those patients were less likely to receive rescue drugs (OR = 0.21, 95% CI = 0.05 to 0.85) than the placebo groups [21]. The only limitation to this meta-analysis is that the authors report a mixed inclusion/exclusion criteria of the studies cited, which may include data on non-migraine headaches, further complicating any conclusions to be drawn. Metoclopramide was also found to produce a statistically significant difference in pain scores at two hours vs sumatriptan, with 59% of patients taking metoclopramide vs 35% taking sumatriptan reporting less pain (difference = 24, 95% CI = 2 to 46, p = .04) at discharge; however, both groups compared equally in taking additional medication for their pain (56% sumatriptan vs 53% metoclopramide) [19]. Thus, the data emphasizes the efficacy of metoclopramide in acute migraine treatment.

The adverse effects associated with metoclopramide, however, can be serious and irreversible. One such effect is tardive dyskinesia, which involves the involuntary movement of the tongue, extremities, and face usually associated with long-term use of antidopaminergic drugs. However, since the expected treatment regimen for acute migraines only involves the ED setting, no such events have been reported [22]. In contrast to other recommended treatments, the adverse effect profile of metoclopramide is less worrisome than that of triptans (hypertensive crisis and myocardial infarction), which is also highly used in the ED setting [23-24]. Studies have also shown that the dose of metoclopramide necessary to achieve 48 hours of sustained freedom from pain was no different in patients receiving 10, 20, or 40 mg; thus, the smallest dose of 10 mg, plus 25 mg of diphenhydramine (dyskinesia prophylaxis), is effective in treating acute migraines [20].

### Metoclopramide vs opioids

Randomized clinical trials (RCT) on opioid vs metoclopramide therapy for acute migraines have not been conducted, despite one study showing that approximately 25% vs 24% of ED choices of therapy included narcotics and antiemetics, respectively. [25]. Therefore, controlled studies comparing the two regimens are crucial.

Griffith, et al. performed a retrospective cohort analysis to assess the reduction in self-reported pain scores, the need for additional rescue medications, adverse effects, and length of ED stay for patients receiving metoclopramide versus hydromorphone [26]. Two hundred individuals selected for the participation were seen in the Northwestern Memorial Hospital ED and treated for migraine headaches between October 2002 and March 2003. A self-reported pain scale of 0-10 was used as an outcome measure. The mean pain score reduction for metoclopramide was significantly greater than hydromorphone (3.7 vs 2.3, p < .01). Patients receiving metoclopramide as the initial abortive therapy were significantly less likely to require the use of additional rescue medications. The use of metoclopramide as the initial abortive therapy was also associated with shorter stays in the ED [26].

## Conclusions

Our narrative review demonstrates that metoclopramide should be used more frequently as the first-line therapy for acute migraine headaches over opioids, with more studies needed to be able to examine the direct comparison with opioids as a treatment for migraines. Although the literature cited does include certain criteria for comparison, such as pain quality, a more thorough analysis should include the outpatient use of these drugs and larger population studies. The use of opioids specifically has been discouraged as migraine treatment by the American Headache Society, citing “insufficient evidence” as the main reason. Metoclopramide, specifically using the 10 mg dose, has been cited as “highly likely to be effective” by the same guidelines. Another major issue with opioids is the growing potential for abuse; therefore, minimizing the use of these drugs for only special circumstances would be beneficial overall. 

## References

[1] Lipton RB, Stewart WF, Diamond S, Diamond ML, Reed M (2001). Prevalence and burden of migraine in the United States: Data from the American Migraine Study II. Headache.

[2] Colman I, Rothney A, Wright SC, Zilkalns B, Rowe BH (2004). Use of narcotic analgesics in the emergency department treatment of migraine headache. Neurology.

[3] Insinga RP, Ng-Mak DS, Hanson ME (2011). Costs associated with outpatient, emergency room and inpatient care for migraine in the USA. Cephalalgia.

[4] Vinson DR (2002). Treatment patterns of isolated benign headache in US emergency departments. Ann Emerg Med.

[5] Mazer-Amirshahi M, Dewey K, Mullins PM, van den Anker J, Pines JM, Perrone J, Nelson L (2014). Trends in opioid analgesic use for headaches in US emergency departments. Am J Emerg Med.

[6] Dowell D, Haegerich TM, Chou R (2016). CDC Guideline for Prescribing Opioids for Chronic Pain - United States, 2016. MMWR Recomm Rep.

[7] Levin M (2014). Opioids in headache. Headache.

[8] Buse DC, Pearlman SH, Reed ML, Serrano D, Ng-Mak DS, Lipton RB (2012). Opioid use and dependence among persons with migraine: results of the AMPP study. Headache.

[9] Ansari H, Kouti L (2016). Drug interaction and serotonin toxicity with opioid use: Another reason to avoid opioids in headache and migraine treatment. Current pain and headache reports. Curr Pain Headache Rep.

[10] Abadie D, Rousseau V, Logerot S, Cottin J, Montastruc JL, Montastruc F (2015). Serotonin syndrome: Analysis of cases registered in the French pharmacovigilance database. J Clin Psychopharmacol.

[11] Dodick D, Freitag F (2006). Evidence-based understanding of medication-overuse headache: clinical implications. Headache.

[12] Klapper JA, Stanton JS (1991). Ketorolac versus DHE and metoclopramide in the treatment of migraine headaches. Headache.

[13] McCarthy LH, Cowan RP (2015). Comparison of parenteral treatments of acute primary headache in a large academic emergency department cohort. Cephalalgia.

[14] Friedman BW, Kapoor A, Friedman MS, Hochberg ML, Rowe BH (2008). The relative efficacy of meperidine for the treatment of acute migraine: a meta-analysis of randomized controlled trials. Ann Emerg Med.

[15] John M. Eisenberg Center for Clinical Decisions and Communications Science (2007). Acute Migraine Treatment in Emergency Settings. Comparative Effectiveness Review Summary Guides for Clinicians.

[16] Scherl ER, Wilson JF (1995). Comparison of dihydroergotamine with metoclopramide versus meperidine with promethazine in the treatment of acute migraine. Headache.

[17] Kelley NE, Tepper DE (2012). Rescue therapy for acute migraine, part 3: opioids, NSAIDs, steroids, and post-discharge medications. Headache.

[18] Desmond PV, Watson KJ (1986). Metoclopramide--a review. Med J Aust.

[19] Friedman BW, Corbo J, Lipton RB, Bijur PE, Esses D, Solorzano C, Gallagher EJ (2005). A trial of metoclopramide vs sumatriptan for the emergency department treatment of migraines. Neurology.

[20] Friedman BW, Mulvey L, Esses D, Solorzano C, Paternoster J, Lipton RB, Gallagher EJ (2011). Metoclopramide for acute migraine: a dose-finding randomized clinical trial. Ann Emerg Med.

[21] Colman I, Brown MD, Innes GD, Grafstein E, Roberts TE, Rowe BH (2004). Parenteral metoclopramide for acute migraine: meta-analysis of randomised controlled trials. BMJ.

[22] Orr SL, Friedman BW, Christie S, Minen MT, Bamford C, Kelley NE, Tepper D (2016). Management of adults with acute migraine in the emergency department: The American Headache Society Evidence Assessment of Parenteral Pharmacotherapies. Headache.

[23] Jensen C, Riddle M (2015). ST-elevation myocardial infarction after sumatriptan ingestion in patient with normal coronary arteries. West J Emerg Med.

[24] Chalaupka FD (2009). Acute myocardial infarction with sumatriptan: a case report and review of the literature. Headache.

[25] Sahai-Srivastava S, Desai P, Zheng L (2008). Analysis of headache management in a busy emergency room in the United States. Headache.

[26] Griffith JD, Mycyk MB, Kyriacou DN (2008). Metoclopramide versus hydromorphone for the emergency department treatment of migraine headache. J Pain.

